# Multilocus sequence typing and biocide tolerance of *Arcobacter butzleri* from Danish broiler carcasses

**DOI:** 10.1186/1756-0500-6-322

**Published:** 2013-08-13

**Authors:** Louise Hesselbjerg Rasmussen, Jette Kjeldgaard, Jens Peter Christensen, Hanne Ingmer

**Affiliations:** 1 Department of Veterinary Disease Biology, University of Copenhagen, Faculty of Health and Medical Sciences, Stigboejlen 4, DK-1870 Frederiksberg, Denmark; 2 Department of Clinical Microbiology, Slagelse Hospital, Slagelse and Roskilde University, Ingemannsvej 18, DK-4200 Slagelse, Denmark; 3 Roskilde University, Universitetsvej 1, P.O. Box 260, DK-4000 Roskilde, Denmark

**Keywords:** *Arcobacter butzleri*, MLST, Chicken slaughterhouse, MIC, Sodium hypochlorite

## Abstract

**Background:**

*Arcobacter* spp. have in recent years received increasing interest as potential emerging enteropathogens and zoonotic agents. They are associated with various animals including poultry and can be isolated from meat products. The possibilities of persistence and cross-contamination in slaughterhouses during meat processing are not well established. We have evaluated the occurrence and persistence of *Arcobacter* spp. in a Danish slaughterhouse and determined the sensitivity of isolates to sodium hypochlorite, a commonly used biocide.

**Results:**

*Arcobacter* contamination was examined in a broiler slaughterhouse by selective enrichment of 235 swabs from the processing line during two production days and after sanitizing in between. In total 13.6% of samples were positive for *A. butzleri* with the majority (29 of 32 isolates) originating from the evisceration machine. No *Arcobacter* spp. was isolated after cleaning. *A. butzleri* isolates confirmed by PCR were typed by multilocus sequence typing (MLST) resulting in 10 new sequence types (STs). Two sequence types were isolated on both processing days. Minimum inhibitory concentration (MIC) to sodium hypochlorite was determined to 0.5% hypochlorite biocide (500 ppm chlorine) for most isolates, which allows growth of *A. butzleri* within the working concentration of the biocide (0.2 - 0.5%).

**Conclusions:**

*A. butzleri* was readily isolated from a Danish broiler slaughterhouse, primarily in the evisceration machine. Typing by MLST showed high strain variability but the recurrence of two STs indicate that some persistence or cross-contamination takes place. Importantly, the isolates tolerated sodium hypochlorite, a biocide commonly employed in slaughterhouse sanitizing, at levels close to the disinfection concentration, and thus, *A. butzleri* may survive the disinfection process although this was not observed in our study.

## Background

*Arcobacter* is member of the family *Campylobacteraceae*[1] and was first isolated in 1977 from aborted bovine and porcine fetuses [2,3]. The genus includes several species of which *A. butzleri, A. cryaerophilus* and to some extent *A. skirrowii* have been considered as animal pathogens implicated in abortions, mastitis and gastrointestinal disorders [4] and as human pathogens causing gastroenteritis and occasionally bacteraemia [5-8]. The genome sequence of *A. butzleri* revealed the presence of homologues of several virulence genes known to contribute to the pathogenesis of *Campylobacter jejuni* but also close resemblance to species of the *Helicobacteraceae*[9]. *Arcobacter* spp. have also been isolated from healthy humans and animals [8,10-12] but in general the prevalence of this emerging pathogen in human disease is likely to be underestimated due to lack of a standard detection procedure [13,14]. Methods for detection and isolation of *Arcobacter* spp. have been suggested and are continuously developing [15-18].

*Arcobacter* spp. have been isolated from a variety of meat products such as beef, pork, poultry and lamb, with notably high prevalence in poultry meat [19-22]. Consistently, poultry seem to be a major reservoir with flock prevalence between 4.3% and 100%, determined with various isolation techniques [23-28]. Some controversies have occurred about the location of *A. butzleri* in broilers, but the intestines and the cloacae seem to be colonized [18,23,29,30]. In poultry slaughterhouses, *Arcobacter* spp. have been isolated from the processing line, process water and carcasses and an increasing prevalence through a slaughter week has been indicated [27,28]. In a Danish study, all of 30 chicken carcasses sampled at a processing plant were positive for *A. butzleri* and six of these were concurrently positive for *A. cryaerophilus*[25]. *A. butzleri* has the ability to attach to surfaces of stainless steel and plastic and is capable of growing at 10°C and forming biofilm [31,32]. These findings suggest that *A. butzleri* may be able to persist in the food processing environment for extended periods of time and contaminate processing equipment despite of sanitizing [28]. In light of this information, we have addressed if *A. butzleri* chicken slaughterhouse isolates tolerate the commonly used biocide sodium hypochlorite in disinfection concentration causing the risk of isolates persisting in this environment. Further, multilocus sequence typing (MLST) of *A. butzleri* isolates was used to evaluate the heterogeneity of the isolates obtained on two consecutive production days and after the sanitizing in between.

## Methods

### Bacterial strains

Reference strains used in this study are listed in Table 1. Bacteria were routinely grown in Brain-Heart-Infusion broth (BHI; OXOID, Greve, Denmark) or *Arcobacter* broth (OXOID) and on *Arcobacter* agar (OXOID; Agar No. 3 incl. *Arcobacter* broth and 50 mL L^-1^ defibrinated calf blood) at 28°C in microaerobic atmosphere generated using CampyGen (OXOID) in sealed jars. For dilution and washing of bacteria peptone-saline water was used (NaCl 9.0 g L^-1^ and peptone 1.0 g L^-1^).

**Table 1:** ***Arcobacter*strains used in this study**

**Species**	**ID**	**Obtained from**
***A. butzleri***	LMG 10828	BCCM/LMG (Ghent, Belgium)
***A. cryaerophilus***	LMG 7536	CCUG (Göteborg, Sweden)
	LMG 10829	BCCM/LMG (Ghent, Belgium)
***A. skirrowii***	LMG 6621	CCUG (Göteborg, Sweden)
***A. trophiarum***	LMG 25534	BCCM/LMG (Ghent, Belgium)
***A. cibarius***	LMG 21996	BCCM/LMG (Ghent, Belgium)
***A. thereius***	LMG 24486	BCCM/LMG (Ghent, Belgium)

### Specifications of the broiler slaughterhouse

A Danish slaughterhouse was selected for an investigation of the occurrence of *Arcobacter* during two processing days. The slaughter capacity was 180,000 chickens per day and the products ranged from whole chickens, breast fillets, wings and chicken legs. After slaughter, scalding, defeathering, evisceration and washing, chickens were conveyed through a 3-hour air chilling step before cutting and packaging. In general, the temperature in the processing areas was 10–12°C. The temperature of the scalding water was 53?±?2°C. The cleaning following directly after each processing day was performed with flusher and foam followed by disinfection. A biocide containing sodium hypochlorite was used for disinfection six days of the week and once per week a quaternary ammonium compound was used. The disinfection biocide containing sodium hypochlorite (Divosan Hypo Plus, JohnsonDiversey, Nivaa, Denmark) was used in a 0.2 – 0.5% working solution, corresponding to 200–500 ppm active chlorine and with a contact time of ten minutes as determined by the manufacturer.

### Sample collection

Samples were collected three times at four sampling points during two consecutive production days (see Table 2 for details). Sterile cotton swabs moistened with *Arcobacter* broth sampled surfaces directly or indirectly in contact with parts of the carcasses. A total of 235 swabs were taken with 55 samples collected from the scalding tank (scalding tank water and overflow), 60 samples from the evisceration machine, 60 samples from the area “cutting of breast fillet” and 60 samples from the area “boning of chicken legs”. *A. butzleri* LMG 10828, *A. cryaerophilus* LMG 7536 and *A. skirrowii* LMG 6621 on agar were used as positive controls and sterile broth samples were used as negative controls. After sampling, swabs were stored individually in 5 mL *Arcobacter* broth in transport tubes under cooled conditions and processed within 30 h.

**Table 2:** **Prevalence of*Arcobacter*spp. in the processing line of a Danish broiler slaughterhouse determined by cultural enrichment and isolation or by direct PCR on enrichment cultures**

	**Processing day 1**		**After cleaning**		**Processing day 2**	
**Place**	Enrichment	PCR	Enrichment	PCR	Enrichment	PCR
**Scalding tank**	0/15	0/15	0/20	0/20	3/20	2/20
**Evisceration machine**	10/20	13/20	0/20	2/20	19/20	17/20
**Area “cutting of breast fillet”**	0/20	0/20	0/20	0/20	0/20	0/20
**Area “boning of chicken leg”**	0/20	2/20	0/20	0/20	0/20	0/20
**Prevalence**	**10/75**	**15/75**	**0/80**	**2/80**	**22/80**	**19/80**
**In percent**	**13.3%**	**20%**	**0%**	**2.5%**	**27.5%**	**23.8%**

### Selective isolation

For selective enrichment favoring *Arcobacter* spp. the samples were added 50 ml L^-1^ defibrinated calf blood and selective supplement comprising of 5-flourouacil (100 mg L^-1^), amphotericin B (10 mg L^-1^), cefoperazone (16 mg L^-1^), novobiocin (32 mg L^-1^) and trimethoprim (64 mg L^-1^) [33] and incubated in 48 h in microaerobic atmosphere at 28°C. The samples were then stored in 15% glycerol at – 80°C until further processing. The samples were thawed in refrigerator at 5°C and aliquots of 250 μL were subcultured individually in 7 mL *Arcobacter* broth, 5% defibrinated calf blood and the selective supplement before incubation for 48 h in microaerobic atmosphere at 28°C. Subsequent 100 μL was inoculated on *Arcobacter* agar with selective supplement and incubated in 48 to 96 h in microaerobic atmosphere at 28°C. Furthermore, the frozen enrichments were subjected to direct PCR identification of *Arcobacter* spp. on crude DNA lysates. After thawing, 300 mL enrichment culture was centrifuged (8.000?×?*g* for 5 min), the supernatant was removed and the pellet was added 300 mL miliQ water. Bacteria were lysed by heating to 99°C for 15 min, cooled down and centrifuged (10.000?×?*g* for 2 min). The supernatants were used as DNA templates in a genus specific PCR amplifying a 181-bp DNA fragment of the 16S rRNA gene from *Arcobacter* spp. [34].

### Species identification by multiplex-PCR

Presumptive *Arcobacter* colonies, determined by colony morphology (<1.5 mm greyish/white colonies) and cell morphology (motile, small, curved rods by microscopy), were subcultured on *Arcobacter* agar and incubated in microaerobic atmosphere at 28°C for 48 h. BHI cultures, grown for 20 h at 28°C, were centrifuged at 11,000?*×?g* for 3 min. Pellets were suspended in 0.5 mL peptone-saline water and centrifuged for 3 min at 14.000 *× g*. DNA was extracted using QIAGEN DNeasy® Blood & Tissue kit following the manufacturer’s instructions with pretreatment for Gram-negative bacteria. All PCR assays were carried out using DreamTaq™ Green PCR Master Mix (2x) (Fermentas Life Sciences) and primers were obtained from TAG Copenhagen (Copenhagen, Denmark). Multiplex-PCR (mPCR) was used for confirmation and identification at species level [35] using a range of *Arcobacter* species as controls (Table 1). Isolates not positive in the mPCR were furthermore evaluated by PCR using primers specific for *A. trophiarum*[36].

### Multilocus sequence typing (MLST)

MLST, a typing method based on partial sequence information of seven housekeeping loci (*aspA, atpA, glnA, gltA, glyA, pgm* and *tkt*), was used for characterization of *A. butzleri* isolates below species level [37]. The MLST gene products were sequenced by Macrogen Inc. (Seoul, Korea) and the sequencing results were processed with CLC Main Work Bench version 5.7.1. To compare with known *A. butzleri* MLST profiles the profiles from the isolates were subjected to the *Arcobacter* specific MLST scheme: [http://pubmlst.org/arcobacter/] [38].

### Phylogenetic analysis

Concatenated *A. butzleri* alleles were aligned using ClustalX [39]. Phylogenetic analyses were conducted using MEGA (Version 5.1; [40]). Un-rooted neighbor-joining phylogenetic trees were constructed with bootstrap analysis (1000 replicates).

### Determination of minimal inhibitory concentration (MIC)

The tolerance towards a slaughterhouse biocide containing sodium hypochlorite (NaClO; Divosan Hypo Plus) was assessed using a standard dilution assay for determination of MIC. The MICs were determined by preparing dilutions starting from the maximal recommended working solution (0.5% and two-fold dilutions from 0.4 to 0.05%) of the biocide in 96-well microtiterplates [41,42]. BHI cultures of the slaughterhouse isolates and *A. butzleri* LMG 10828, grown for 20 h at 28°C, were diluted in BHI broth to give absorbance at 600 nm (*A*_600_) of 0.01 and were inoculated to biocide broth dilutions to a total volume of 200 μL per well. Positive (no NaClO) and negative (no inoculum) growth controls were included and the experiment was performed with quadruple determinations. The lowest NaClO concentration to inhibit bacterial growth after 20 h of incubation at 28°C in microaerobic atmosphere was considered as the MIC. To verify that inhibition from the slaughterhouse biocide was caused by the active ingredient, NaClO, the experiment was repeated with NaClO from Sigma-Aldrich (Broendby, Denmark; active chlorine; 4.00 - 4.99%).

## Results and discussion

### Occurrence of *Arcobacter* in a Danish slaughterhouse

A Danish chicken slaughterhouse, with a capacity of 180,000 chickens per day, was subjected to swab sampling on selected sites over two consecutive processing days during production, and after the sanitizing in between, to evaluate the *Arcobacter* contamination. In total, 32 of 235 (13.6%) samples were confirmed positive for *A. butzleri* by multiplex PCR after selective enrichment [33,43] but none of the samples taken on clean surfaces after sanitizing were positive. The majority of the isolates (29 of 32) were recovered from the evisceration machine; hence 48.3% of the samples from this site were positive. The distribution in the processing line is shown in Table 2. Using a direct PCR-technique, 36 of 235 (15.3%) samples were found positive for *Arcobacter* spp. and by this method, two samples from the meat processing area (cutting and boning) were positive as well as two samples from the evisceration machine after cleaning, which were not found in the selective enrichment. Otherwise, *Arcobacter* positive samples were identical using the two methods, except for three additional positives in the evisceration machine (Day 1) and two ‘false negative’ samples from the evisceration machine (Day 2) that were disqualified due to unspecific bands on agarose gels. The high prevalence of *A. butzleri* in the evisceration area supports earlier reports of *Arcobacter* positive chicken gut samples [23,26,28] and suggests that contamination is likely to originate from the chicken gut.

*A. butzleri* was the only species isolated. This may be due to the enrichment procedure, as controls of *A. cryaerophilus* and *A. skirrowii* failed to grow under the enrichment conditions. Enrichment culturing has previously been shown to favor outgrowth of *A. butzleri*[33], but variations in the enrichments culturing used in this study (prolonged cooled storage before enrichment and inclusion of a freeze storage stage of enriched samples) have likely had a negative effect on the survival of *Arcobacter* isolates, especially non-*A. butzleri* species [29]. This is supported by the difference in prevalence of *Arcobacter* on the first and second sampling day, as the prevalence was twice as high in samples from the second day, which had a shorter storage time. The *A. butzleri*, *A. cryaerophilus* and *A. skirrowii* controls were all detected using direct PCR. Interestingly, we detected *Arcobacter* in two out of 20 swabs from the evisceration machine after cleaning, but these were not isolated using selective plating. *Arcobacter* spp. have previously been recovered from cleaned processing machines in poultry slaughterhouses, even after a three-day break in the production, as well as from slaughtered broilers before evisceration [27]. Furthermore, *A. butzleri* is able to multiply at the temperature present in slaughterhouses (10°C) and to form biofilm on stainless steel surfaces [31], which suggest that *A. butzleri* may be able to establish itself in the slaughterhouse environment. In consequence, we investigated the heterogeneity of *A. butzleri* isolated from chicken carcasses and slaughterhouse equipment by MLST and the ability of the obtained isolates to tolerate disinfection with the routinely used biocide (sodium hypochlorite).

### Characterization of *A. butzleri* isolates by multilocus sequence typing (MLST) and phylogeny

Typing of *Arcobacter* by MLST was demonstrated to be useful for discrimination below species level of isolates of *A. butzleri, A. cryaerophilus, A. skirrowii* and *A. cibarius* and a database for *Arcobacter* MLST profiles are available online [http://pubmlst.org/arcobacter/] [37,38]. We performed MLST analysis of 32 *A. butzleri* isolates obtained in the chicken slaughterhouse, resulting in identification of 10 new sequence types (STs) comprising both known alleles and 13 new alleles (Table 3). No known STs were identified. The relatively high numbers of new alleles and STs show the heterogeneity of the *A. butzleri* isolates, but also reflects the limited number of profiles in the *Arcobacter* MLST database. With three isolates on the first sampling day and 12 on the second sampling day, ST 367 was found to be the most prevailing (47% of isolates, see Table 4). Furthermore, ST 367 (15 isolates) and ST 373 (five isolates) were isolated during both production days in the evisceration machine. The two STs 367 and 373 have no identical alleles and it seems more likely that the reoccurrence of these STs are due to cross-contamination rather than identical sequence types being present at different broiler flock farms.

**Table 3:** **Overview of MLST results of*A. butzleri*LMG 10828 and slaughterhouse isolates with allelic profiles according to the*Arcobacter*MLST database**[37]

**Isolation area**	**Allelic profile**							**ST**
	***aspA***	***atpA***	***glnA***	***gltA***	***glyA***	***pgm***	***tkt***	
**Evisceration machine**	153	4	11	11	177	102	11	367
**Evisceration machine**	5	12	122*	15	36	86	2	368
**Evisceration machine**	30	34	9	30	144	35	175*	369
**Scalding tank**	206*	143*	2	37	166	53	162	370
**Evisceration machine**	14	49	26	55	449*	102	176*	371
**Evisceration machine**	23	44	24	14	450*	101	20	372
**Evisceration machine**	205*	144*	123*	55	447*	233*	177*	373
**Evisceration machine**	5	5	5	19	120	11	65	374
**Evisceration machine**	20	25	7	2	354	32	2	375
**Evisceration machine**	175	25	41	19	88	101	14	376

* New allele.

**Table 4:** **Occurrence and isolation site of 32*Arcobacter butzleri*isolates assigned ten different MLST sequence types (ST)**

	**Day 1**		**Day 2**	
**ST**	**No. of isolates**	**Area**	**No. of isolates**	**Area**
367	3	Evisceration	12	Evisceration
368	1	Evisceration	-	-
369	1	Evisceration	-	-
370	-	-	3	Scalding tank
371	-	-	2	Evisceration
372	-	-	1	Evisceration
373	4	Evisceration	1	Evisceration
374	1	Evisceration	-	-
375	-	-	1	Evisceration
376	-	-	2	Evisceration

**Figure 1:**
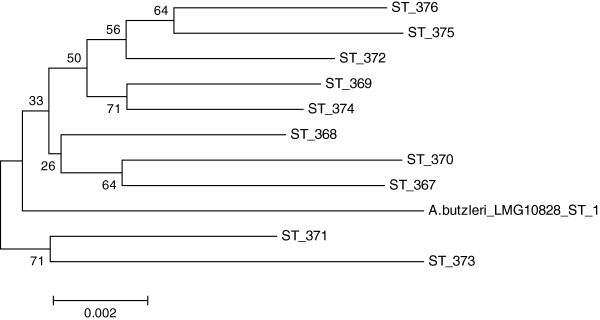
**Neighbour-joining phylogenetic tree of concatenated *A. butzleri *alleles from ST 367 to ST 376 (and including *A. butzleri *LMG 10828 ST 1) isolated at a Danish chicken slaughterhouse.** Bootstrap analysis with 1000 replications was performed, based on 3,341 bp, and bootstrap values are shown at the nodes.

Isolation of *A. butzleri* from the scalding water during production on the second sampling day suggests a risk of cross-contamination within a production period. However, *Arcobacter* spp. are unlikely to survive more than few minutes in the scalding water (53?±?2°C) [44] and the ST found in scalding water was not found elsewhere in the production. Phylogenetic analysis of the concatenated *A. butzleri* alleles was conducted to evaluate possible clustering within the isolates. As established with the MLST, the strain diversity is high and in conjunction with the relatively low number of isolates this results in a phylogenetic tree (Figure 1) that is not very robust (low bootstrap values) and shows no clustering of isolates. Arcobacters have previously been characterized by molecular typing using ERIC-PCR and RAPD-PCR [45,46], by MLST [9] and by AFLP and PFGE profiling [47]. In general, these molecular fingerprinting techniques show a high discrimination of strains due to considerable heterogeneity of *Arcobacter*[15]. A great advantage of MLST genotyping is the online available database of *Arcobacter* spp. isolates collected worldwide [38] and thereby the possibility to compare results. Yet, so far it has not been possible to relate specific STs with hosts or geographical areas [37].

### Biocide tolerance

In the slaughterhouse, cleaning is performed on a daily basis by foaming all surfaces with a detergent and high pressure cleaning with hot water. Following cleaning, disinfection is performed with a biocide containing sodium hypochlorite (NaClO/Chlorine) at concentrations of 5–15%. The working solution of the biocide is 0.2 – 0.5% corresponding to 200–500 ppm active chlorine and the contact time is approximately 10 min. To address the susceptibility of *A. butzleri* to sodium hypochlorite we determined the minimum inhibitory concentration (MIC) by a standard broth dilution method used in similar studies [41,42]. We found that the majority of arcobacters were only inhibited by the sodium hypochlorite containing biocide in the maximum recommended working solution; 0.5% corresponding to 500 ppm active chlorine. *A. butzleri* LMG 10828 had a MIC of 0.5% sodium hypochlorite, as did 31 of the 32 slaughterhouse isolates (97%) (data not shown). The last slaughterhouse isolate and the remaining *Arcobacter* type strains (Table 1) had a MIC of 0.2% corresponding to 200 ppm active chlorine. This tolerance was confirmed using reagent grade sodium hypochlorite (Sigma-Aldrich). During regular cleaning operations the biocides are allowed a contact time of 10 min when applied for disinfection. Here we determine that the lower working concentration (0.2%) of sodium hypochlorite does not have a lethal effect on *A. butzleri* even after 20 h of exposure. Despite the biocide tolerance of *Arcobacter* isolates we did not obtain any isolates in the slaughterhouse after cleaning and disinfection with chlorine containing detergent, although two of a total of 80 swabs taken after sanitizing were *Arcobacter* positive by direct PCR. This suggests that the combined cleaning and disinfection procedures are generally working satisfactorily, but in highly contaminated areas it might not be sufficient.

We isolated *A. butzleri* almost exclusively from the evisceration area in the chicken slaughter house. Previous studies report findings of arcobacters in the intestines of chickens [23,48] and increased prevalence of *A. butzleri* on neck skin after evisceration with identical genotypes in the intestines and on the carcasses within a flock [28]. These findings suggest that arcobacter contamination occurs at the evisceration step and that contamination from the intestinal content to the carcass surface takes place. We determined a high biocide tolerance of the *A. butzleri* isolates obtained from the slaughterhouse, illustrating that *A. butzleri* can grow at concentrations close to the working solution of NaClO and we confirmed the presence of *Arcobacter* in two swab samples taken after sanitizing. Considering the disinfection contact time of 10 min, it is likely that *A. butzleri* may survive sanitizing. This assumption is supported by the isolation of two cases of identical STs of *A. butzleri,* determined by MLST, reoccurring on two consecutive production days, suggesting either persistence or continuous re-contamination of *A. butzleri.*

## Conclusions

The MLST genotyping of *A. butzleri,* isolated on two consecutive production days in a chicken slaughterhouse, showed high heterogeneity of the isolates but nonetheless reoccurrence of two sequence types on both production days. We further show that *A. butzleri* can survive and multiply in prolonged exposure (20 h) to working concentrations of a commonly used disinfecting biocide, sodium hypochlorite. Thus, *A. butzleri* may be able to persist in the slaughterhouse after disinfection.

## Competing interests

The authors declare that they have no competing interests.

## Authors’ contributions

LHR conceived the research idea and performed the laboratory work, analyzed the data and wrote the manuscript. JK helped in sample collection, analyzing and interpreting data and preparation of the manuscript. JPC and HI helped in designing the study and supervision of the work and revised the manuscript. All authors read and approved the final manuscript.

## References

[B1] Vandamme P Deley J Proposal for a new family, *Campylobacteraceae* Int J Syst Bacteriol 1991 41 451 455 10.1099/00207713-41-3-451

[B2] Ellis WA Neill SD O'Brien JJ Ferguson HW Hanna J Isolation of *Spirillum/Vibrio*-like organisms from bovine fetuses Vet Rec 1977 100 451 452 10.1136/vr.100.21.451 560078

[B3] Ellis WA Neill SD O'Brien JJ Hanna J Isolation of *Spirillum*-like organisms from pig fetuses Vet Rec 1978 102 106 416554 10.1136/vr.102.5.106-a

[B4] Vandamme P Vancanneyt M Pot B Mels L Hoste B Dewettinck D Polyphasic taxonomic study of the emended genus *Arcobacter* with *Arcobacter butzleri* comb-nov and *Arcobacter skirrowii* sp-nov, an aerotolerant bacterium isolated from veterinary specimens Int J Syst Bacteriol 1992 42 344 356 10.1099/00207713-42-3-344 1503968

[B5] Vandamme P Pugina P Benzi G Van Etterijck R Vlaes L Kersters K Outbreak of recurrent abdominal cramps associated with *Arcobacter butzleri* in an Italian school J Clin Microbiol 1992 30 2335 2337 1400998 10.1128/jcm.30.9.2335-2337.1992 PMC265502

[B6] On SLW Stacey A Smyth J Isolation of *Arcobacter butzleri* from a neonate with bacteremia J Infect 1995 31 225 227 8586843 10.1016/s0163-4453(95)80031-x

[B7] Yan JJ Ko WC Huang AH Chen HM Jin YT Wu JJ *Arcobacter butzleri* bacteremia in a patient with liver cirrhosis J Formos Med Assoc 2000 99 166 169 10770033

[B8] Vandenberg O Dediste A Houf K Ibekwem S Souayah H Cadranel S *Arcobacter* species in humans Emerg Infect Dis 2004 10 1863 1867 10.3201/eid1010.040241 15504280 PMC3323243

[B9] Miller WG Parker CT Rubenfield M Mendz GL Wösten MM Ussery DW The complete genome sequence and analysis of the Epsilonproteobacterium *Arcobacter butzleri* PLoS One 2007 2 e1358 10.1371/journal.pone.0001358 18159241 PMC2147049

[B10] Wesley IV Wells SJ Harmon KM Green A Schroeder-Tucker L Glover M Fecal shedding of *Campylobacter* and *Arcobacter* spp. in dairy cattle Appl Environ Microbiol 2000 66 1994 2000 10.1128/AEM.66.5.1994-2000.2000 10788372 PMC101445

[B11] Óngör H Cetinkaya B Acik MN Atabay HI Investigation of arcobacters in meat and faecal samples of clinically healthy cattle in Turkey Lett Appl Microbiol 2004 38 339 344 10.1111/j.1472-765X.2004.01494.x 15214736

[B12] Houf K Stephan R Isolation and characterization of the emerging foodborn pathogen *Arcobacter* from human stool J Microbiol Methods 2007 68 408 413 10.1016/j.mimet.2006.09.020 17097175

[B13] Prouzet-Mauleon V Labadi L Bouges N Menard A Megraud F *Arcobacter butzleri*: Underestimated enteropathogen Emerg Inf Dis 2006 12 307 309 10.3201/eid1202.050570 PMC3373082 16494760

[B14] Engberg J On SLW Harrington CS Gerner-Smidt P Prevalence of *Campylobacter*, *Arcobacter*, *Helicobacter,* and *Sutterella* spp. in human fecal samples as estimated by a reevaluation of isolation methods for Campylobacters J Clin Microbiol 2000 38 286 291 10618103 10.1128/jcm.38.1.286-291.2000 PMC88711

[B15] Gonzalez I Garcia T Fernandez S Martin R Current status on *Arcobacter* research: An update on DNA-based identification and typing methodologies Food Anal Method 2012 5 956 968 10.1007/s12161-011-9343-9

[B16] Lehner A Tasara T Stephan R Relevant aspects of *Arcobacter* spp. as potential foodborne pathogen Int J Food Microbiol 2005 102 127 135 10.1016/j.ijfoodmicro.2005.03.003 15982771

[B17] Van Driessche E Houf K Van Hoof J De Zutter L Vandamme P Isolation of *Arcobacter* species from animal feces FEMS Microbiol Lett 2003 229 243 248 10.1016/S0378-1097(03)00840-1 14680706

[B18] Eifert JD Castle RM Pierson FW Larsen CT Hackney CR Comparison of sampling techniques for detection of *Arcobacter butzleri* from chickens Poult Sci 2003 82 1898 1902 14717547 10.1093/ps/82.12.1898

[B19] Kabeya H Maruyama S Morita Y Ohsuga T Ozawa S Kobayashi Y Prevalence of *Arcobacter* species in retail meats and antimicrobial susceptibility of the isolates in Japan Int J Food Microbiol 2004 90 303 308 10.1016/S0168-1605(03)00322-2 14751685

[B20] Rivas L Fegan N Vanderlinde P Isolation and characterisation of *Arcobacter butzleri* from meat Int J Food Microbiol 2004 91 31 41 10.1016/S0168-1605(03)00328-3 14967558

[B21] Scullion R Harrington CS Madden RH A comparison of three methods for the isolation of *Arcobacter* spp. from retail raw poultry in Northern Ireland J Food Prot 2004 67 799 804 15083734 10.4315/0362-028x-67.4.799

[B22] Lee MH Cheon DS Choi S Lee BH Jung JY Choi C Prevalence of *Arcobacter* species isolated from retail meats in Korea J Food Prot 2010 73 1313 1316 20615344 10.4315/0362-028x-73.7.1313

[B23] Ho HTK Lipman LJA Gaastra W The introduction of *Arcobacter* spp. in poultry slaughterhouses Int J Food Microbiol 2008 125 223 229 10.1016/j.ijfoodmicro.2008.02.012 18579247

[B24] Atanassova V Kessen V Reich F Klein G Incidence of *Arcobacter* spp. in poultry: quantitative and qualitative analysis and PCR differentiation J Food Prot 2008 71 2536 10.4315/0362-028x-71.12.2533 19244910

[B25] Atabay HI Waino M Madsen M Detection and diversity of various *Arcobacter* species in Danish poultry Int J Food Microbiol 2006 109 139 145 10.1016/j.ijfoodmicro.2006.01.020 16516995

[B26] Kabeya H Maruyama S Morita Y Kubo M Yamamoto K Arai S Distribution of *Arcobacter* species among livestock in Japan Vet Microbiol 2003 93 153 158 10.1016/S0378-1135(02)00312-7 12637003

[B27] Houf K De Zutter L Van Hoof J Vandamme P Occurrence and distribution of *Arcobacter* species in poultry processing J Food Prot 2002 65 1233 1239 12182473 10.4315/0362-028x-65.8.1233

[B28] Houf K De Zutter L Verbeke B Van Hoof J Vandamme P Molecular characterization of *Arcobacter* isolates collected in a poultry slaughterhouse J Food Prot 2003 66 364 369 12636286 10.4315/0362-028x-66.3.364

[B29] Van Driessche E Houf K Discrepancy between the occurrence of *Arcobacter* in chickens and broiler carcass contamination Poult Sci 2007 86 744 751 17369548 10.1093/ps/86.4.744

[B30] Ho H Lipman L Gaastra W The presence of *Arcobacter* species in breeding hens and eggs from these hens Poult Sci 2008 87 2404 2407 10.3382/ps.2008-00092 18931194

[B31] Kjeldgaard J Jørgensen K Ingmer H Growth and survival at chiller temperatures of *Arcobacter butzleri* Int J Food Microbiol 2009 131 256 259 10.1016/j.ijfoodmicro.2009.02.017 19297052

[B32] Assanta MA Roy D Lemay MJ Montpetit D Attachment of *Arcobacter butzleri*, a new waterborne pathogen, to water distribution pipe surfaces J Food Prot 2002 65 1240 1247 12182474 10.4315/0362-028x-65.8.1240

[B33] Houf K Devriese LA De Zutter L Van Hoof J Vandamme P Development of a new protocol for the isolation and quantification of *Arcobacter* species from poultry products Int J Food Microbiol 2001 71 189 196 10.1016/S0168-1605(01)00605-5 11789937

[B34] González I García T Antolín A Hernández PE Martín R Development of a combined PCR-culture technique for the rapid detection of *Arcobacter* spp. in chicken meat Lett Appl Microbiol 2000 30 207 212 10.1046/j.1472-765x.2000.00696.x 10747252

[B35] Douidah L De Zutter L Vandamme P Houf K Identification of five human and mammal associated *Arcobacter* species by a novel multiplex-PCR assay J Microbiol Methods 2010 80 281 286 10.1016/j.mimet.2010.01.009 20096309

[B36] De Smet S Vandamme P De Zutter L On SLW Douidah L Houf K *Arcobacter trophiarum* sp. nov., isolated from fattening pigs Int J Syst Evol Microbiol 2011 61 356 361 10.1099/ijs.0.022665-0 20305065

[B37] Miller WG Wesley IV On SLW Houf K Megraud F Wang GL First multi-locus sequence typing scheme for *Arcobacter* spp BMC Microbiol 2009 9 196 10.1186/1471-2180-9-196 19751525 PMC2755481

[B38] Miller WG Arcobacter MLST Database 2012 http://pubmlst.org/arcobacter/

[B39] Larkin MA Blackshields G Brown NP Chenna R McGettigan PA McWilliam H Clustal W and Clustal X version 2.0 Bioinform 2007 23 2947 2948 10.1093/bioinformatics/btm404 17846036

[B40] Tamura K Peterson D Peterson N Stecher G Nei M Kumar S MEGA5: Molecular evolutionary genetics analysis using maximum likelihood, evolutionary distance, and maximum parsimony methods Mol Biol Evol 2011 28 2731 2739 10.1093/molbev/msr121 21546353 PMC3203626

[B41] Mavri A Kurincic M Mozina SS The prevalence of antibiotic and biocide resistance among *Campylobacter coli* and *Campylobacter jejuni* from different sources Food Technol Biotechnol 2012 50 371 376

[B42] Sheridan À Lenahan M Duffy G Fanning S Burgess C The potential for biocide tolerance in *Escherichia coli* and its impact on the response to food processing stresses Food Control 2012 26 98 106 10.1016/j.foodcont.2012.01.018

[B43] Houf K Tutenel A De Zutter L Van Hoof J Vandamme P Development of a multiplex PCR assay for the simultaneous detection and identification of *Arcobacter butzleri, Arcobacter cryaerophilus* and *Arcobacter skirrowii* FEMS Microbiol Lett 2000 193 89 94 10.1111/j.1574-6968.2000.tb09407.x 11094284

[B44] Van Driessche E Houf K Survival capacity in water of *Arcobacter* species under different temperature conditions J Appl Microbiol 2008 105 443 451 10.1111/j.1365-2672.2008.03762.x 18298536

[B45] Houf K De Zutter L Van Hoof J Vandamme P Assessment of the genetic diversity among arcobacters isolated from poultry products by using two PCR-based typing methods Appl Environ Microbiol 2002 68 2172 2178 10.1128/AEM.68.5.2172-2178.2002 11976086 PMC127564

[B46] Atabay HI Bang DD Aydin F Erdogan HM Madsen M Discrimination of *Arcobacter butzleri* isolates by polymerase chain reaction-mediated DNA fingerprinting Lett Appl Microbiol 2002 35 141 145 10.1046/j.1472-765X.2002.01152.x 12100590

[B47] Gonzalez A Ferrus MA Gonzalez R Hernandez J Molecular fingerprinting of *Campylobacter* and *Arcobacter* isolated from chicken and water Int Microbiol 2007 10 85 90 17661285

[B48] Atabay HI Corry JEL The prevalence of *Campylobacters* and *Arcobacters* in broiler chickens J Appl Microbiol 1997 83 619 626 10.1046/j.1365-2672.1997.00277.x 9418023

